# Diagnostic accuracy of the breast MRI Kaiser score in suspected architectural distortions and its comparison with mammography

**DOI:** 10.1038/s41598-023-50798-7

**Published:** 2024-01-03

**Authors:** Ozge Aslan, Aysenur Oktay

**Affiliations:** Department of Radiology, Ege University Faculty of Medicine, 35100 Bornova, Izmir State Turkey

**Keywords:** Cancer, Oncology, Cancer, Magnetic resonance imaging, Radiography

## Abstract

Suspicious architectural distortion is an elusive finding in breast cancer diagnosis. This study aimed to evaluate the diagnostic accuracy of the Kaiser score for suspicious architectural distortions observed on mammography and compare it with the BI-RADS score of the lesion. Mammograms performed between January 2013 and March 2023 were retrospectively analyzed for the presence of suspicious architectural distortion. Forty-one patients, who had at least 1 year of radiological follow-up or pathology results, and underwent breast MRI, were included in the study. Mammography findings and the BI-RADS category of the lesion were assessed. MRI findings were evaluated and Kaiser scoring was performed according to the tree flowchart. Ninety-one percent of the enhanced lesions had a Kaiser score of 5 and above. In the diagnosis of malignancy, the Kaiser score yielded an accuracy of 75.61% (AUC 0.833). A statistically significant correlation was observed indicating that a malignant diagnosis was more prevalent in patients with a Kaiser score of 5 and above (*p* < 0.05). Additionally statistically significant relationship was also observed between the BI-RADS category of architectural distortions on mammography and the Kaiser score (*p* = 0.007). The combined utilization of mammography findings and the evidence-based Kaiser score in suspected architectural distortions provides more accurate results in the differential diagnosis of breast cancer.

## Introduction

Architectural distortion is characterized by the pulling of breast tissue towards the center and disruption of the normal structure, without the presence of a mass in the breast tissue^1^. Architectural distortion is a subtle mammographic finding that has a high positive predictive value for malignancy. It ranks as the third most common finding in breast cancer cases^2^. Architectural distortion can be associated with both benign and malignant conditions. The positive predictive value for malignancy is reported to be 75% and was the most common finding missed in false negative mammograms^2,3^. Benign conditions that can cause architectural distortion include scar tissue resulting from surgery, radial scar and complex sclerosing lesion, sclerosing adenosis, fat necrosis, fibromatosis, and granular cell tumor.

Architectural distortion can be observed either independently or in combination with microcalcifications, asymmetry, and masses. In such cases, the presence of architectural distortion raises a high suspicion for malignancy. Compared to the early diagnosis of breast cancer through microcalcifications, early detection of architectural distortions has a greater impact on improving patient prognosis^4^.

If architectural distortion is suspected on mammography, further evaluation is typically conducted using ultrasonography (US) and categorized as BI-RADS 4. Tissue diagnosis is then obtained through a core needle biopsy guided by ultrasound. If an ultrasound equivalent is not available, MRI can be utilized as a problem-solving method. If architectural distortion is detected on MRI, a tissue diagnosis can be obtained through an MRI-guided biopsy.

MRI is considered the most sensitive method for diagnosing breast cancer, and it has specific indications for cancer staging. These indications include disease detected in the premenopausal period, dense breast structure, suspicion of multicentric lesions, and the presence of invasive lobular carcinoma type^5^. Additionally, MRI, with its high negative predictive value, serves as a valuable problem-solving tool in cases where suspicious architectural distortions cannot be conclusively evaluated through mammography and US^5–7^. The BI-RADS score on MRI provides a standard and structured reporting language for lesion identification, but does not provide a decision rule to guide diagnosis^6^. Evaluation without a clear rule can be challenging, as it relies on the experience of the evaluator, which may lead to different decisions being made^5^. For this purpose, the Kaiser Score, developed by Werner Alois Kaiser, offers a framework for evaluating breast lesions based on four independent diagnostic criteria: lesion border features, kinetic enhancement curve type, internal enhancement, and presence of edema. The scoring system, implemented through a three-step tree flowchart, indicates the likelihood of malignancy and serves as a clinical decision rule in determining the possibility of malignancy^6^. As a result of the evaluation, scores ranging from 1 to 11 indicate an increased probability of malignancy. Lesions with a Kaiser score below 5 can generally be considered benign, while patients with a Kaiser score of 5 or higher would typically require a biopsy for further evaluation^6,8^. Suspicion of malignancy is particularly high, especially in scores exceeding 7, and patients with benign biopsy pathology results should be carefully evaluated. The Kaiser score has been reported to reduce interobserver differences and false positives^6,9^. Studies have demonstrated that unnecessary biopsies and surgeries can be reduced in patients with true negative results by utilizing the recommendations based on the BI-RADS classification and Kaiser score^8,10^. The relationship between achitectural distortion in mammography and Kaiser score has not been investigated, yet.

In this study, the purpose was to calculate the diagnostic accuracy of the Kaiser score for suspicious architectural distortions observed in mammography, and to compare it with the BI-RADS category of mammography.

## Material and methods

**Figure 1:**
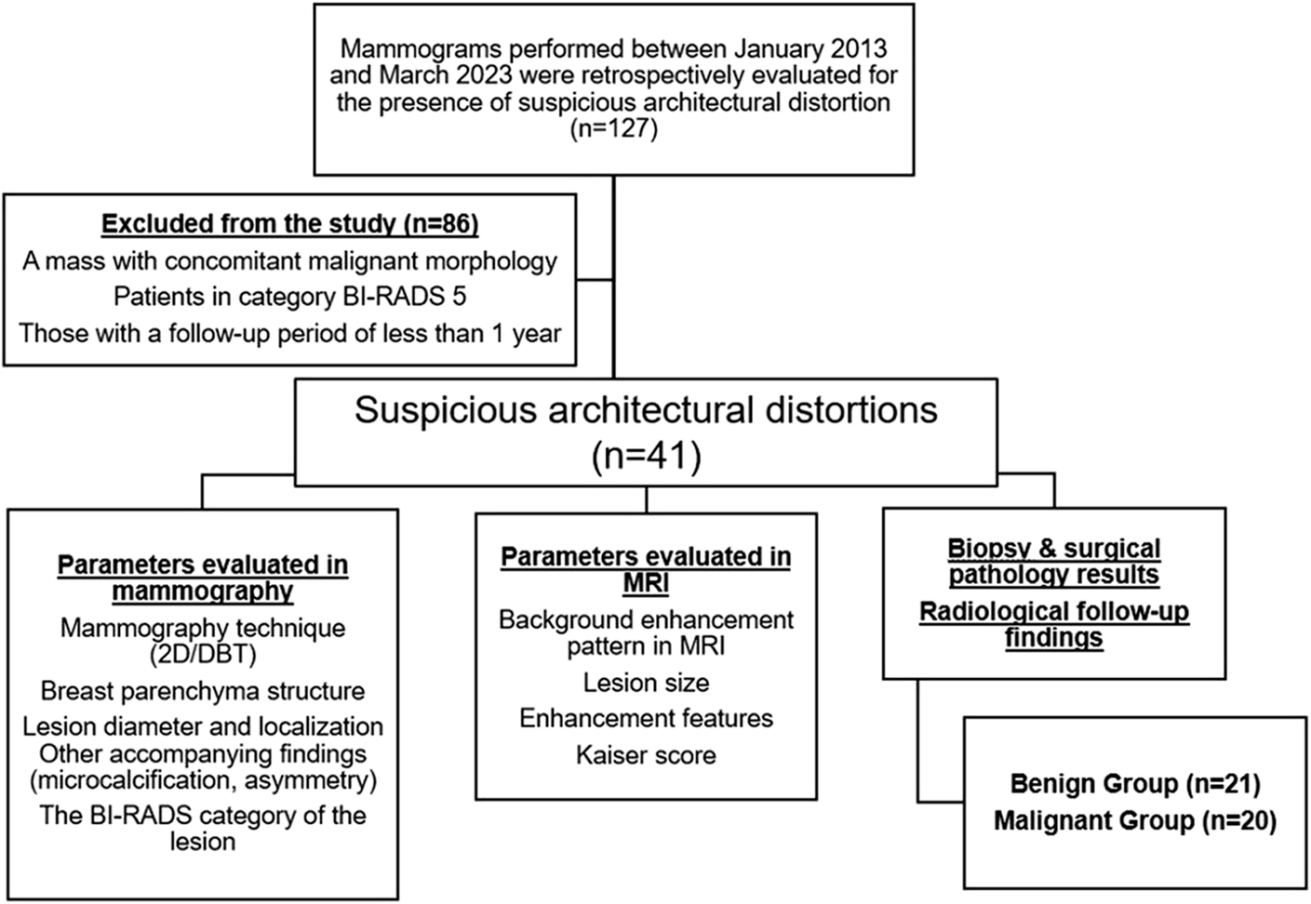
Flowchart of the study.

The mammography reports between January 2013 and March 2023 were reviewed, and the patients with the finding of architectural distortion classified as BI-RADS 4 category were retrospectively evaluated from the picture archiving and communication system (PACS) of our hospital (Sectra IDS7 Workstation, Sectra AB, Sweden) (n = 127). Patients classified as BI-RADS 5 with a concurrent mass exhibiting malignant characteristics and patients who had a radiological follow-up period of less than 1 year were excluded from the study (n = 86). Forty-one patients, aged between 18 and 80, who had at least 1 year of radiological follow-up or pathology results, and underwent breast MRI, were included in the study (Fig. 1).

The radiological images were reviewed by two radiologists with 5 years and 33 years of breast radiology experience, blinded to the pathology, imaging reports and each other. If any conflict occured, the final decision between two observers was reached by consensus.

### Imaging

Full-field digital mammograms (FFDM) were conducted using either the Selenia Dimensions system by Hologic or the Amulet Innovality system by Fujifilm. The mammograms were performed in the standard 2D craniocaudal (CC) and mediolateral oblique (MLO) positions. Standard craniocaudal (CC) mammograms and digital breast tomosynthesis (DBT) mediolateral oblique (MLO) radiographs were acquired in patients with dense parenchymal structure. Additionally, synthetic MLO radiographs were generated.

The MRIs were conducted using a 1.5 Tesla MR imaging unit (Magnetom Amira and Symphony, Siemens Healthineers, Erlangen, Germany) and a 3-Tesla MR imaging unit (Magnetom Verio, Siemens Healthineers, Erlangen, Germany). The patients were positioned prone, and their breasts were placed within a dedicated surface breast coil. The MR images were acquired using the following sequences: axial, fat-suppressed, and fast spin-echo T2-weighted imaging sequence, as well as pre-contrast and post-contrast dynamic axial T1-weighted three-dimensional, fat-suppressed, fat-spoiled gradient-echo sequence. For the contrast-enhanced sequences, rapid bolus injection of gadopentetate dimeglumine (Magnevist; Bayer, Berlin, Germany) was administered at a dosage of 0.1 mmol/kg of body weight, followed by a 10-ml saline flush at a rate of 2 mL/s through an indwelling intravenous catheter. Multiplanar reformation, thin slab maximal intensity projection (MIP) images and rotating MIP images were created from the first high-resolution post-contrast “peak” phase. Kinetic enhancement curves were performed by placing a region of interest on suspicious enhancing lesions using  picture archiving and communication system (PACS) (Sectra IDS7 Workstation, Sectra AB, Sweden) .

### Data collection and analysis

Patient age, mammography technique (2D/DBT), breast parenchyma structure, MG findings (lesion size, localization, presence of other accompanying findings such as microcalcifications or asymmetry), and the BI-RADS (Breast Imaging Reporting and Data Systems) category of the lesion were assessed. MG and MRI findings were evaluated according to the ACR (American College of Radiology) BI-RADS atlas 5th Edition^10,11^. Lesions classified in the BI-RADS 4 category were further categorized based on the current suspicion of malignancy. They were classified as 4A (low suspicion), 4B (moderate suspicion), or 4C (high suspicion) categories on mammograms. In the statistical analysis of the BI-RADS classification, those with 4A were considered low, and those with 4B and 4C were considered highly suspicious for malignancy.

**Figure 2:**
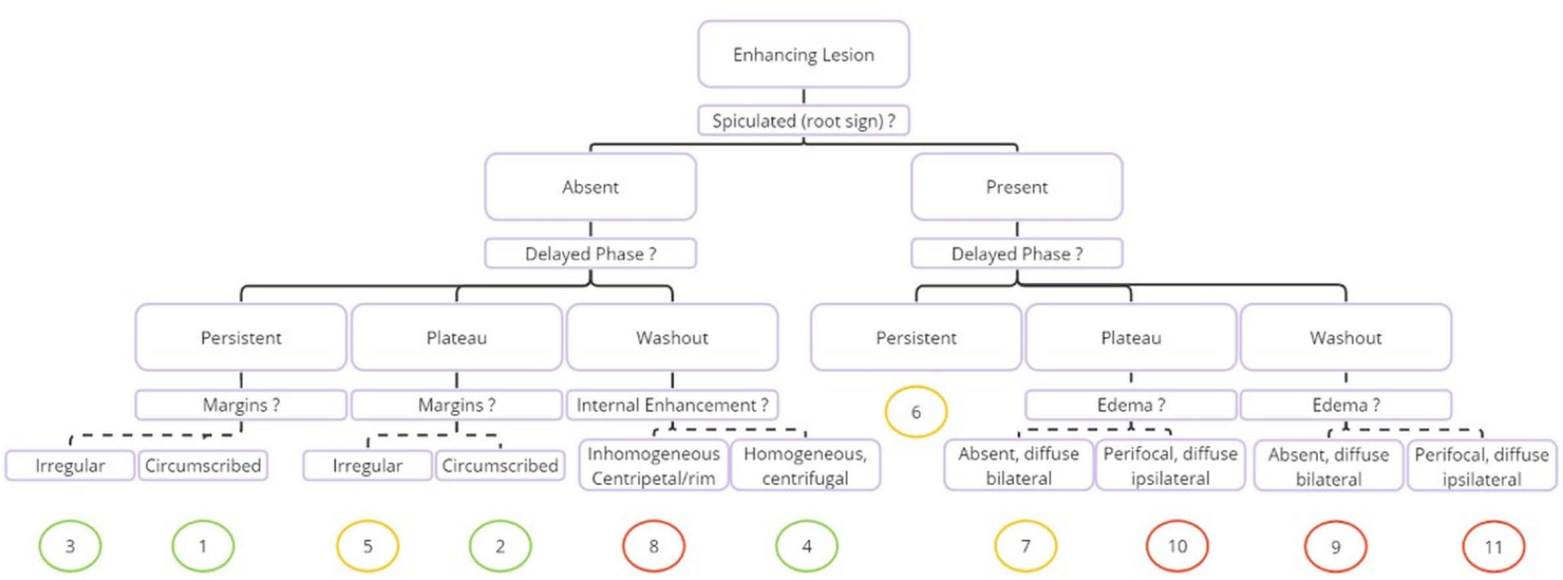
The tree flowchart of Kaiser score (References^6,12^).

In dynamic contrast enhanced breast MRI, the background enhancement pattern, if there is an enhanced lesion, the lesion diameter, the enhancement features (enhancement type, shape, lesion border features, internal enhancement pattern, kinetic curve type) were evaluated. Additionally, features such as the presence of spiculation, kinetic curve type, lesion margin characteristics, internal enhancement pattern, and the presence of edema in contrast-enhanced lesions on MRI were assesed for Kaiser score according to the tree flowchart, which can be accessed online at http://www.meduniwien.ac.at/kaiser-score/^6,12^. The Kaiser score was determined to be between 1 and 11 (Fig. 2). Those with a Kaiser score of 5 or more were considered to be suspicious for malignancy, and those with a score below 5 were regarded as benign.

Biopsy or surgical histopathology results, if available, and the duration of radiological follow-up were documented. Lesions that did not enhance in MRI and remained stable during radiological follow-ups, along with patients with benign pathology results, constituted the benign group. Patients with malignant pathology results formed the malignant group. Diffusion-weighted images (DWI) were excluded from the analysis due to the unavailability in all patients.

### Statistical analysis

Descriptive statistics of the data were given as mean, standard deviation, median, minimum, maximum, frequency, and percentage values. The normality assumption of the quantitative data was checked using the Shapiro–Wilk test. The independent sample t-test was used for the normally distributed variables, while the Mann–Whitney U test was used for the variables that did not meet the normality assumption. The correlation of quantitative data with each other was evaluated using Spearman’s Rho correlation coefficient. Cohen’s kappa correlation was used for the aggrement of data. Kaiser score 4 was accepted as the cut-off value, and sensitivity, specificity, positive predictive value, negative predictive value and accuracy were calculated. The relationships between categorical variables were examined using the Pearson Chi-square test. The diagnostic accuracy of the Kaiser score was evaluated using ROC analysis, and the area under the curve (AUC) was calculated. Intraclass correlation coefficient (ICC) was used to match the numerical MRI Kaiser scores between two radiologists.

Statistical analyses were performed using the IBM SPSS Statistics 25.0 (IBM SPSS Statistics for Windows, Version 25.0. Armonk, NY: IBM Corp.) software package. The level of significance was set at 0.05 for all analyses.

### Ethics approval and consent to participate

The study was performed per the ethical standards as laid down in the 1964 Declaration of Helsinki. Ethics committee approval of this study was obtained from the medical research ethics committee of our hospital (Ege University Faculty of Medicine). Ethics committee approval decision number: 23–4.1 T/31.

## Results

The mean age of the patients was 51.5 years (± 8.66 standard deviation) (Table 1). A total of 22 patients underwent 2D FFDM, while 19 patients underwent DBT. The most frequently observed breast pattern was type C, with a rate of 61%. When considering the mammographic findings (Table 2), architectural distortions were predominantly located in the right breast (63%).

**Table 1:** Numerical measurements and results.

	Age (years)	Lesion size on mammography (mm)	Lesion size on MRI (mm)	Kaiser score (1st Radiologist)	Kaiser score (2nd Radiologist)	The duration of radiological follow-up (months)
Mean	51.54	17.22	19.88	5.37	5.41	20.78
Standard deviation	8.661	7.532	17.497	3.08	3.09	15.920
Median	51	15	17	6	6	14
Minimum	33	6	0	3	3	7
Maximum	68	50	85	11	10	60

**Table 2:** Mammography and ultrasonography findings.

Mammography findings		n	%
Breast paranchyma pattern	Type B	8	19.5
	Type C	25	61
	Type D	8	19.5
Lesion side	Right breast	26	63
	Left Breast	15	37
Accompanying finding	No additional findings	21	51
	Microcalcification	2	5
	Asymmetry	18	44
The BI-RADS category of the lesion	4A	11	27
	4B	22	54
	4C	8	19
Ultrasonography findings	No finding	12	29
	Posterior acoustic shadowing	4	10
	Architectural distortion	14	34
	Mass	2	5
	Non mass (altered echotexture)	9	22

Architectural distortion was the sole finding on mammography in 51% (n = 21) of the patients. In two patients, a cluster of microcalcifications was present as an accompanying finding. Asymmetry accompanying architectural distortion was observed in 44% (n = 18).

On mammography, the lesions were categorized into three groups as BI-RADS 4A, 4B, and 4C, with the most frequent category being BI-RADS 4B (54%).

The mean diameter of architectural distortions on mammography was 17.22 mm (± standard deviation 7.5 mm) (Table 1). No finding was detected in 29% of the patients on US examination. The most common US finding was architectural distortion (34%) (Table 2).

BI-RADS classification of architectural distortions on mammography has a sensitivity of 90%, specificity of 42.86% and accuracy of 65.85% in distinguishing between malignant and benign lesions (Table 3).

**Table 3:** BI-RADS category and Kaiser score statistical analyzes results.

	BI-RADS category		Kaiser score	
	Value (%)	95% CI (%)	Value (%)	95% CI (%)
Sensitivity	90	68.30–98.77	100	83.16–100
Specificity	42.86	21.82–65.98	52.38	29.78–74.29
Positive likelihood ratio	1.58	1.06–2.35	2.10	1.34–3.29
Negative likelihood ratio	0.23	0.06–0.95	0	–
Positive predictive value (PPV)	48.78	50.18–69.08	66.67	56.08–75.80
Negative predictive value (NPV)	81.82	52.49–94.83	100	71.51–100
Accuracy	65.85	49.41–79.92	75.61	59.70–87.64

When considering the MRI findings, the mean lesion diameter was 19.8 mm (± standard deviation 17.5 mm) (Table 1). No pathological enhancement was observed in MRI for 8 patients (Table 4). These patients remained stable during the radiological follow-up and were included in the benign group. The most frequent enhancement pattern observed on MRI was non-mass enhancement (46%). The lesions exhibited homogeneous, inhomogeneous, and clustered nodular enhancement features, with the most common type being the inhomogeneous pattern, observed in 21 cases. The most frequent kinetic curve was type 1 (48.5%).

**Table 4:** Magnetic resonance ımaging (MRI) findings.

MRI findings		n	%
Enhancement pattern	No enhancement	8	20
	Mass	14	34
	Non mass	19	46
Enhancement type	Homogeneous	8	24
	Inhomogeneous	21	64
	Clustered nodular	4	12
Kinetic curve type	Persistent	16	49
	Plateau	9	27
	Washout	8	24
Kaiser score	< 5	11	27
	≥ 5–11	30	73

**Figure 3:**
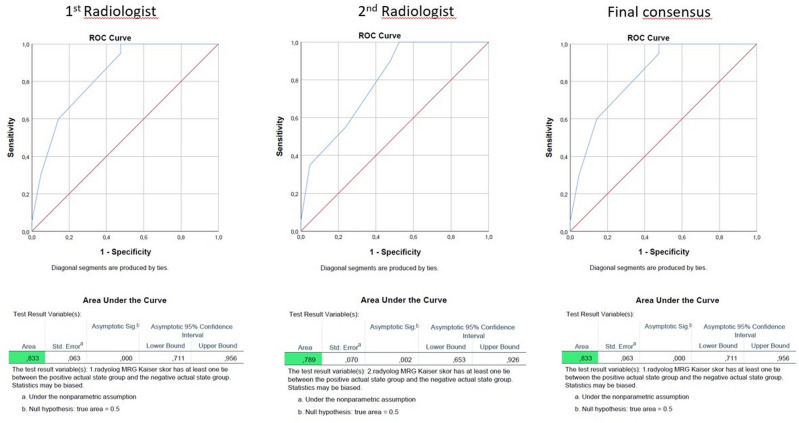
Kaiser Score diagnostic accuracy analyzes based on ROC (Receiver Operating Characteristic).

According to the first radiologist's evaluation the mean Kaiser score value was 5.37, with a minimum of 3 and a maximum of 11. When considering the diagnostic accuracy of the Kaiser score for differentiating between benign and malignant lesion, the sensitivity was 100%, the specificity was 52.38%, the positive predictive value (PPV) was 66.67%, the negative predictive value (NPV) was 100%, and the overall accuracy was 75.61%. The area under the curve (AUC) was calculated to be 0.833 according to the ROC (Receiver Operating Characteristic) analysis (Fig. 3). According to the second radiologist's evaluation the mean Kaiser score value was 5.41, with a minimum of 3 and a maximum of 10. When considering the diagnostic accuracy of the Kaiser score, the sensitivity was 100%, the specificity was 47.62%, PPV was 64.52%, NPV was 100%, and the overall accuracy was 73.17%. AUC was calculated to be 0.789 (Fig. 3). A conflict occured between two observers in only one patient which was solved by consensus. The patient in whom the conflict arose was in scores of Kaiser 4 and 5. The consensus in that patient was Kaiser 4, and the final histopathological diagnosis was lobular carcinoma in situ and radial scar.

**Figure 4:**
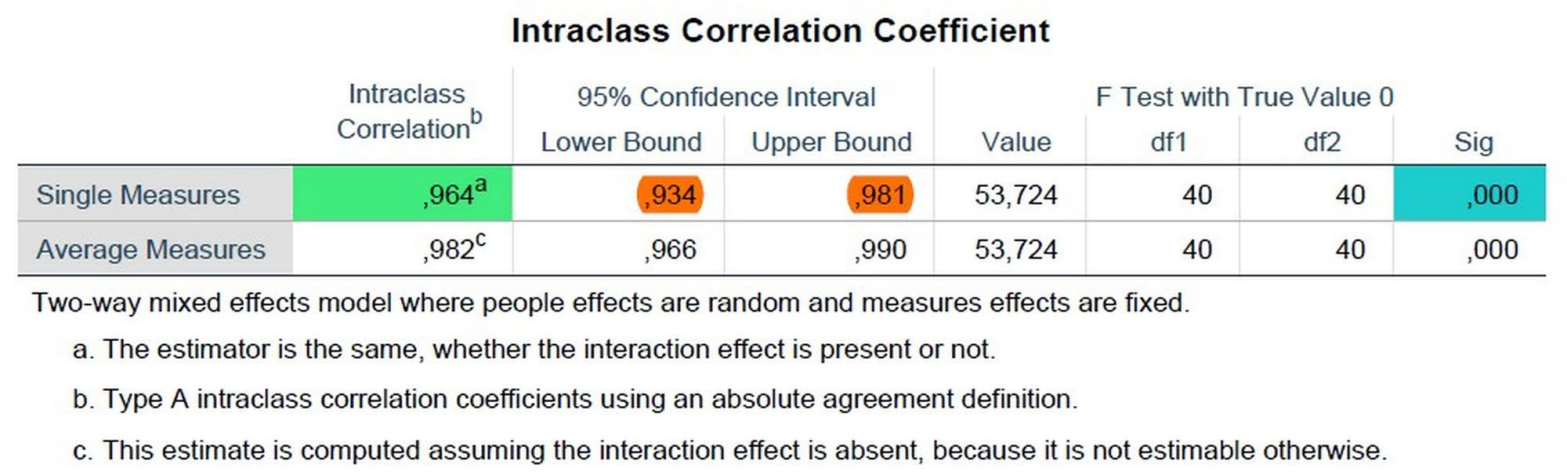
Intraclass correlation coefficient results.

Intraclass correlation coefficient (ICC) was used to match the numerical MRI Kaiser scores between two radiologists. The coefficient was 0.964 (0.934—0.981), regarded as perfect agreement (Fig. 4).

Among all contrast-enhanced lesions, 91% of them had a Kaiser score of 5 or above. These lesions were classified as BI-RADS 4B and 4C categories on mammography. Statistical significance was found between BI-RADS classification and Kaiser score evaluation in mammography (*p* = 0.007) (Table 5).

**Table 5:** Relationship between Kaiser score and BI-RADS category.

	BI-RADS category		*p*
	4A	4B &4C	
Kaiser score			
< 5	6	5	0.007
≥ 5	5	25	

Out of the 41 patients, 10 were monitored through radiological follow-up, while 31 patients underwent surgical excision (Table 6). The mean duration of radiological follow-up was 20.8 months (± 15.9 months) (Table 1).

**Table 6:** Surgical excision and radiological follow-up results.

Follow-up results			n	%
Stable on radiological follow-up			10	24
Surgically excised	Benign (n:11%27)	Atypical ductal hyperplasia + Atypical intraductal papilloma	1	2.4
		Atypical ductal hyperplasia + Radial scar	1	2.4
		Radial scar	5	12
		Radial scar + Intraductal papilloma	1	2.4
		Radial scar + Lobular carcinoma in situ	1	2.4
		Sclerosing adenosis	2	5
	Malignant (n:20%49)	Ductal carcinoma in situ	2	5
		Invasive ductal carcinoma	7	17
		Invasive lobular carcinoma	4	10
		Invasive lobular carcinoma + Lobular carcinoma in situ	3	7
		Myoepithelial carcinoma + Ductal carcinoma in situ	1	2.4
Total			41	100

**Figure 5:**
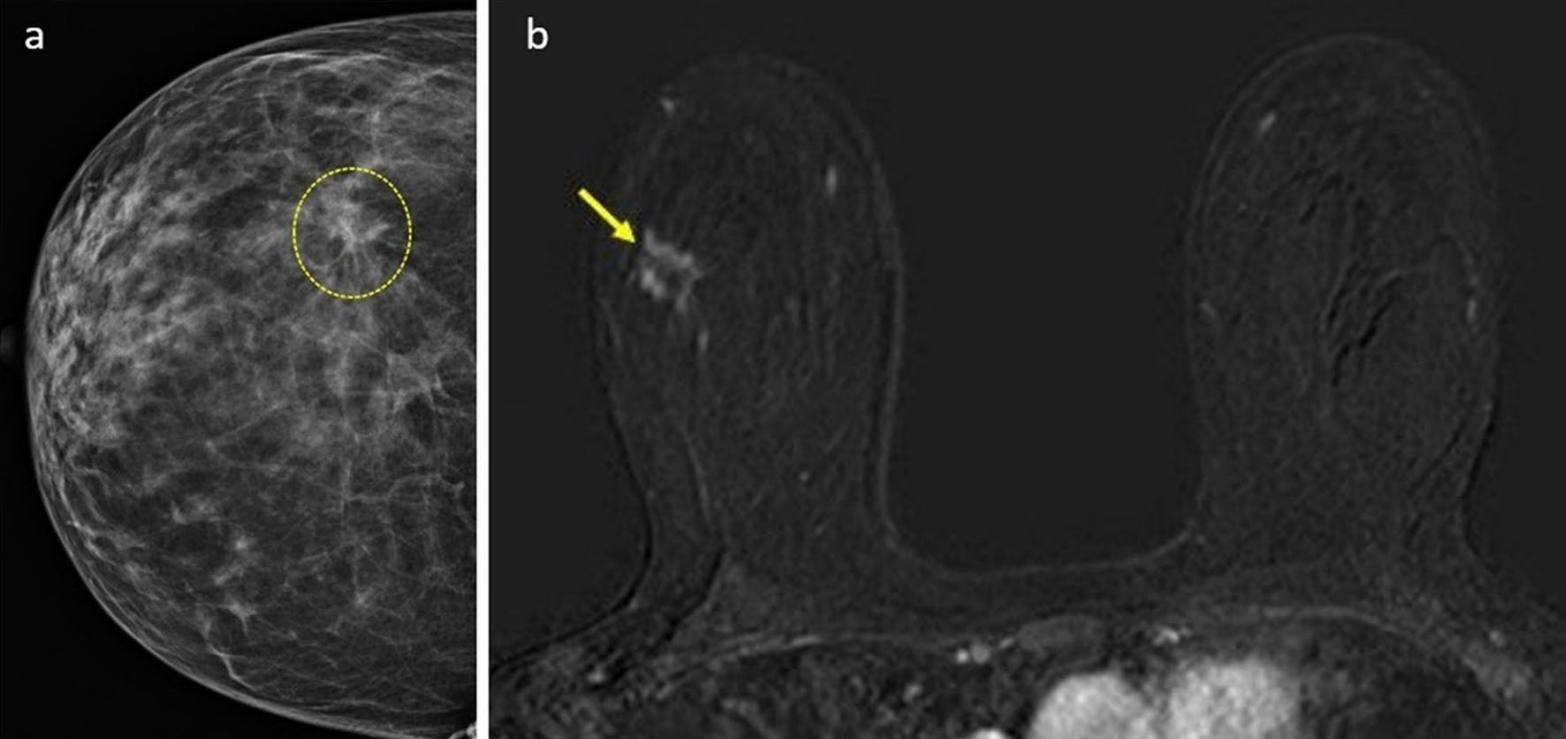
A 49-year-old woman underwent mammography for routine screening, revealing a suspicious area of architectural distortion (indicated by dashed yellow lines) in the outer quadrant of the right breast (**a**), in the area where distortion was observed on mammography, a lesion with irregular margins, rim-like, and inhomogeneous enhancement was identified on the dynamic contrast-enhanced subtraction MRI (**b**). The Kaiser score of the lesion was 7. Surgical excision pathology confirmed the diagnosis of invasive ductal carcinoma.

**Figure 6:**
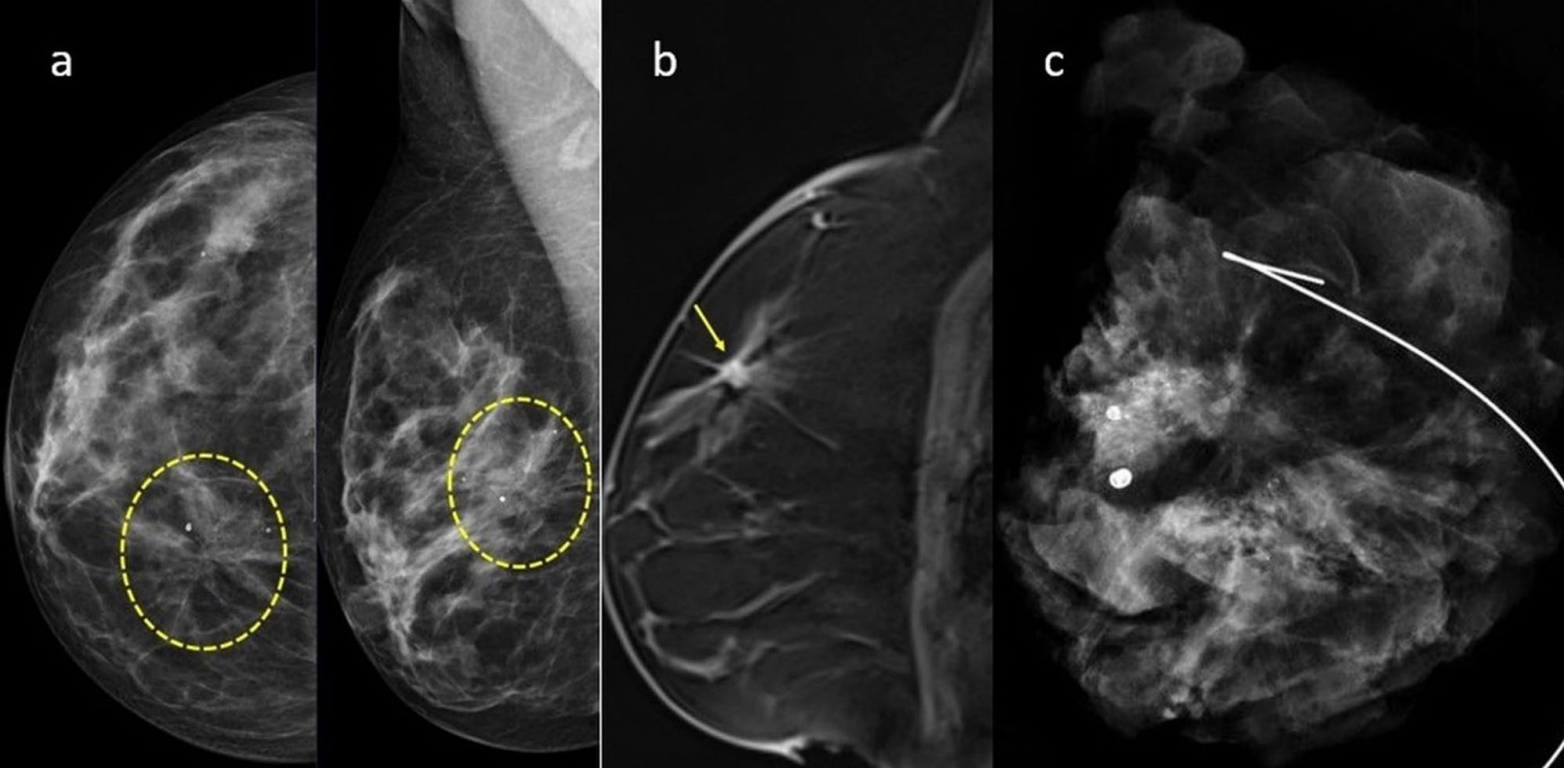
A 48-year-old female underwent a follow-up mammogram, revealing an area of architectural distortion (indicated by dashed yellow lines) in the upper inner quadrant of the right breast, which includes microcalcifications (BI-RADS4B category) (**a**), on the T1weighted dynamic contrast-enhanced sagittal MR image; it appears as a mass lesion with irregular borders and spicular extension to the periphery in the upper quadrant of the right breast (**b**). The Kaiser score of the lesion is 7, the specimen graphy reveals the removal of the area of architectural distortion containing microcalcifications (**c**). The surgical pathology result confirms the presence of atypical ductal hyperplasia and radial scar.

Among the patients who underwent surgical excision, 11 were found to have benign lesions, while 20 patients were diagnosed with malignant tumors. There were two cases of in situ cancers in the malignant group, while the remaining cases were invasive cancer (Fig. 5). High-risk breast lesions (atypical ductal hyperplasia [ADH], atypical intraductal papilloma [AIP], lobular carcinoma in situ [LCIS], radial scar [RS]), were identified in 9 patients who had a Kaiser score of 5 or above, indicating a high suspicion of malignancy (Fig. 6). However these lesions were also included in the benign group. Among the patients diagnosed with high-risk lesions, only 3 of them had a Kaiser score of 7 or higher, while the rest had a score of 6. In this study, the false positivity of the Kaiser score arose from nine high-risk breast lesions and two sclerosing adenosis categorized as histopathologically benign.

Based on the statistical analysis, a significant correlation was found between the diameter of the architectural distortion and the BI-RADS category (*p* = 0.020). A significant correlation was discovered between the presence of accompanying findings on mammography and the BI-RADS category of the lesion (*p* = 0.020). Additionally, a significant correlation was identified between the size of the architectural distortion on mammography and the size of the enhanced lesion on MRI (*p* = 0.028).

It was shown that, as the diameter of the architectural distortion increased in mammography and MRI, the rate of malignancy detection also increased (*p* = 0.048). A malignant diagnosis was more frequently observed in patients with a Kaiser score of 5 and above (*p* < 0.05) (Table 7).

**Table 7:** Relationship between Kaiser score & BI-RADS category and malignancy.

	Benign n (%)	Malignant n (%)	*p*
Kaiser score (1st radiologist)			
< 5	11 (52.4)	0 (0)	< 0.001
≥ 5	10 (47.6)	20 (100)	
Kaiser score (2nd radiologist)			
< 5	10 (47.6)	0 (0)	0.001
≥ 5	11 (52.4)	20 (100)	
BI-RADS category			
4A	9 (42.9)	2 (10)	0.018
4B&4C	12 (57.1)	18 (90)	

## Discussion

Architectural distortion is among the non-palpable findings associated with early-stage breast cancer. Early detection improves the prognosis of the patient. In breast MRI, which is employed as a problem-solving method, evaluating more parameters based on evidence with the Kaiser score provides greater clinical guidance. In this study, the diagnostic accuracy of the Kaiser score was determined to be 75.61%. The area under the curve (AUC) was calculated to be 0.833 according to the ROC analysis, as shown in Fig. 3. This indicates a reasonably good discriminatory power for the diagnostic accuracy of the Kaiser score.

Wang et al. have reported a high diagnostic accuracy of the Kaiser score in their study, with an area under the curve (AUC) of 0.958^7^. The disparity of our findings with Wang’s study could be attributed to the scope of our study only focusing on architectural distortions and also the exclusion of lesions classified in the BI-RADS5 category.

The difference in accuracy between our study and the literature may be due to the inclusion of high-risk breast lesions (ADH, RS, LCIS, etc.) with a Kaiser score above 5 in the benign group. In our study, the false positivity of the Kaiser score arises from nine high-risk breast lesions categorized as histopathologically benign. However, as indicated in the literature, these lesions mimic malignancy on radiological imaging and their excision is recommended due to the potential risk of accompanying malignancy^13^. The other two lesions in the benign group were diagnosed as sclerosing adenosis, but their Kaiser scores were determined as 6 by both radiologists. Sclerosing adenosis usually occurs during routine imaging and screenings in the premenopausal period or in biopsies performed for other reasons^14^. There are a limited number of studies on MRI findings of sclerosing adenosis^14^. Additionally, there is no publication regarding the specific Kaiser score for sclerosing adenosis.

In this study, where there was high agreement between radiologists, Kaiser scores were found to have different cut off values in only one patient. This patient was also in the group of high-risk lesions diagnosed with LCIS and RS.

There is no other study in the literature that specifically evaluates the Kaiser score in relation to only architectural distortions and high-risk breast lesions. By incorporating the Kaiser score into a radiomics model, it is possible to develop a predictive model to estimate the probability of malignancy in high-risk breast lesions. Performing radiological follow-up in appropriate patients with high-risk breast lesions, who have a low Kaiser score and radiology-pathology compatibility, can help reduce unnecessary surgeries.

While the Kaiser score has not been incorporated into the flowchart yet, it is suggested that including diffusion-weighted imaging findings in the scoring system would offer an additional quantitative advantage. According to the scoring system, lesions with a Kaiser score above 4 can be downgraded to 4 points if they exhibit high apparent diffusion coefficient (ADC) values^6^. Nevertheless, if the quality of the ADC map is deemed to be low, it is recommended to exclude ADC measurements from the scoring system to avoid the potential risk of false negativity. Due to the limited availability of DWI in our patient cohort, ADC values were not included in the analysis. However, in two of our patients who were diagnosed with high-risk lesions (ADH and RS), DWI was performed, and high signal intensity was observed in the corresponding area on ADC maps. When their Kaiser scores were downgraded accordingly, they dropped below 5, and it was observed that the utilization of DWI findings would further enhance diagnostic accuracy.

In addition, there is a suggestion to add 2 points to the scoring system when a low Kaiser score is observed in low-grade ductal carcinoma in situ cases accompanied by suspicious microcalcifications^6^. Wengert et al. have demonstrated that the use of the Kaiser score in suspicious microcalcifications can lead to a reduction of unnecessary biopsies in 63.5% of cases^15^. In our study, a statistically significant relationship was found between the Kaiser score and the BI-RADS classification on mammography.

Marino et al. demonstrated that the variability between the Kaiser scoring system and the readers, which arises from differences in reader experience, can be minimized^16^. In our study, perfect agreement was found between the Kaiser scores of the two radiologists. Furthermore, since the parameters utilized in the scoring system are independent of the imaging protocols, it proves to be a valuable tool in daily practice, particularly during radiology training and the early stages of breast imaging implementation.

The most significant limitations of this study are its retrospective design and the relatively small number of patients. Furthermore, due to the absence of DWI in some patients, ADC maps could not be included in the statistical analysis. More comprehensive results can be obtained through future studies that incorporate DWI, the Kaiser score, and artificial intelligence models, employing prospective planning with larger patient cohorts.

## Conclusions

The combined utilization of mammography findings and the evidence-based MRI Kaiser score in suspected architectural distortions provides more accurate results in the differential diagnosis of breast cancer. The Kaiser score can serve as a valuable tool in treatment decision-making and follow-up planning, helping to mitigate potential discrepancies arising from varying levels of experience.

## Data Availability

The data used in the study are responsibly managed by the researchers in accordance with individual privacy protection guidelines, as permitted by our ethics committee. The datasets generated during and analysed during the current study are available from the corresponding author on reasonable request.
